# Prognostic factors for patients treated with abiraterone

**DOI:** 10.2144/fsoa-2019-0079

**Published:** 2019-12-12

**Authors:** Cecília M Alvim, André Mansinho, Rita S Paiva, Raquel Brás, Patrícia M Semedo, Soraia Lobo-Martins, Carolina B da Ponte, Daniela Macedo, Leonor Ribeiro, José P dos Reis, Isabel Fernandes, Luís Costa

**Affiliations:** 1 Medical Oncology Department, Hospital de Santa Maria, Centro Hospitalar Universitário Lisboa Norte, Lisbon, 1649-035, Portugal; 2 Luís Costa Lab, Instituto de Medicina Molecular, Faculdade de Medicina, Universidade de Lisboa, 1649-028 Lisbon, Portugal; 3 Urology Department, Hospital de Santa Maria, Centro Hospitalar Universitário Lisboa Norte, Lisbon, 1649-035, Portugal

**Keywords:** abiraterone acetate, metastatic castration-resistant prostate cancer, prostate-specific antigen response

## Abstract

**Aim:**

To evaluate prostate-specific antigen response (PSAr) defined as a ≥50% decrease in PSA concentration from the pretreatment value, as a prognostic factor in patients with metastatic castration-resistant prostate cancer (mCRPC) treated with abiraterone acetate (AA).

**Methods:**

Retrospective evaluation of patients with mCRPC treated with AA.

**Results:**

124 patients were identified. Median overall survival and progression-free survival for patients achieving PSAr versus patients without PSAr were 29.3 versus 9.7 months and 17.0 versus 5.2 months, respectively. Multivariate analysis confirmed that PSAr correlated with better overall survival (hazard ratio: 0.19; 95% CI: 0.10−0.38; p < 0.001) and progression-free survival (hazard ratio: 0.24; 95% CI: 0.14−0.41; p < 0.001).

**Conclusion:**

PSAr can be utilized as prognostic and predictive factors in mCRPC patients treated with AA.

## Lay abstract

Therapeutic options for metastatic castration-resistant prostate cancer are expanding. However, biomarkers with predictive and prognostic value to assist in treatment guidance are lacking. The aim of this study was to evaluate prostate-specific antigen response as a prognostic factor in patients treated with abiraterone acetate. It was possible to confirm that prostate-specific antigen response correlated with better overall survival and progression-free survival in this patient cohort, evidencing the possible role of biochemical markers as surrogates for response and prognosis, especially in diseases that are difficult to evaluate radiographically.

Prostate cancer is the most common malignancy in men and the second leading cause of death from cancer [1]. Most metastatic prostate cancer patients show an initial favorable response with androgen-deprivation therapy, but castration resistance inevitably develops in the majority [2]. Evidences suggest that most patients with metastatic disease develop castration-resistant prostate cancer (CRPC) within 5 years of follow-up, and median survival from development of castration resistance is approximately 30 months [3,4]. Treatment of mCRPC has evolved over the last decade, with results from large randomized clinical trials leading to the approval of several new agents showing an overall survival (OS) benefit in patients with mCRPC, both pre- and post-chemotherapy-based regimens [2,4,5]. One of these agents is abiraterone acetate (AA), an oral inhibitor of the CYP450 c17, a critical enzyme in the extragonadal and testicular synthesis, resulting in undetectable serum testosterone concentration [3,6].

The increasing availability of new agents poses the problem of choosing the right treatment for the right patient in the correct timing, being particularly relevant the identification of predictive and prognostic factors that allow for an individual therapeutic strategy and estimation of expected benefit [3,6–8]. However, it is important to acknowledge that, although AA is effective in both pre- and post-chemotherapy setting, discrepancies exist regarding its effectiveness, with only a fraction of patients actually benefiting in the long term. The cumulative introduction of agents like docetaxel, AA and enzalutamide (another second-generation antiandrogen) elicits the development of resistance, emphasizing the need for biomarkers for patients who are candidates for new-generation hormonal agents as AA [7,8].

Prostate-specific antigen (PSA) is widely used to monitor prostate cancer and its decline after chemotherapy has been acknowledged as a valid surrogate for OS and progression-free survival (PFS) at 3 months [9–13]. Retrospective studies confirmed that patients with mCRPC with a 50% decline in PSA from baseline have a survival benefit compared with patients who do not achieve such magnitude of reduction. However, the role of PSA as a surrogate predictor for OS in the course of treatment with new-generation hormonal agents and after chemotherapy remains uncertain [9–11,14].

The aim of the present retrospective analysis was to evaluate PSA response as a prognostic factor in patients treated with AA. The proposed hypothesis is that PSA response is able to identify patients more likely to benefit from AA treatment and who will survive longer.

## Methods

### Study population

Patients with mCRPC treated with AA, both pre- and post-docetaxel, at Hospital de Santa Maria − Centro Hospitalar Universitário Lisboa Norte between January 2013 and December 2017, were consecutively included and retrospectively evaluated. In the predocetaxel setting, patients were asymptomatic or minimally symptomatic, with no need for opiate analgesia. In the postdocetaxel setting, patients had confirmed progression or intolerable toxicity under chemotherapy treatment. All patients with confirmed bone metastases were under antiresorptive therapy, either with denosumab or zoledronate.

The study's primary end point was the correlation of PSA response to AA treatment − defined as a ≥50% decrease in PSA concentration from the pretreatment baseline value (which was confirmed in a second PSA evaluation) − with OS and PFS. Secondary end points included the association of OS and PFS with other clinical and laboratory baseline characteristics retrieved from patients’ clinical records whenever available: age, Gleason score, disease sites, previous docetaxel therapy, primary tumor treated, performance status (Eastern Cooperative Oncology Group [ECOG]), hemoglobin, LDH, ALP and total PSA. Additionally, the following data were retrieved: time of PSA response to AA, time of disease progression, time with androgen blockade (androgen-deprivation therapy) to mCRPC, time of AA discontinuation and time of death or last follow-up visit.

During AA treatment, patients were monthly evaluated for PSA values. Radiographic assessment with computed tomography or Tc^99^ bone scan was performed whenever biochemical or clinical progression was suspected. Progression and treatment response were defined according to the Prostate Cancer Clinical Trials Working Group 2 (PCWG2) criteria.

### Statistical analysis

Sample size was not preplanned, as this was a convenience sample, only determined by the predefined inclusion criteria.

Patients were stratified according to each prognostic factor included in the analysis: Gleason score (≥8 and <8), performance status (0 or 1 vs ≥2), visceral metastases (yes/no), hemoglobin levels (≥12 and <12 g/dl), LDH (≥median and <median), ALP (≥upper limit of normal and <upper limit of normal), total PSA (≥median and <median), PSA response (yes/no), primary tumor treated (yes/no) and previous docetaxel treatment (yes/no). Median and interquartile range (IQR) were reported for continuous variables, and frequencies for categorical variables. To compare categorical variables among groups, χ^2^ and Fisher exact tests were used whenever appropriate. The Mann–Whitney test was used to compare medians.

Outcome measures were PFS and OS. PFS was defined as time between AA therapy's start and time of progression, as established by PCWG2 criteria. OS was defined as time between AA therapy's start and death, irrespective of its cause. Median OS and PFS were obtained using the Kaplan–Meier method and log-rank test was used to compare group outcomes. To assess the effect of analyzed parameters in the duration of response and prognosis, univariate and multivariate analyses were conducted using Cox regression model. All statistical tests were two tailed and statistical significance was assumed when p > 0.05. IBM's SPSS statistics v.24 was used for statistical analysis.

## Results

A total of 124 patients were treated with AA during the nearly 5-year time period considered. All patients had sufficient follow-up information and were therefore eligible for this analysis.

### Baseline characteristics

Median age of the cohort was 73.4 years (IQR: 67.1−80.0), with 41.9% of patients with Gleason score ≥8 and 91.1% of patients with ≤1 ECOG performance score. In total, 46.8% of patients received treatment for the primary tumor (M0 at diagnosis) and 37.9% had been previously treated with docetaxel. The median time to mCRPC diagnosis was 52.0 months (IQR: 17.0−78.0), with 22 patients (17.7%) having visceral metastases, with bone being the most frequent (93.5%) metastization site. Concerning prognostic factors at baseline, median values of hemoglobin, LDH, ALP and total PSA were 12.6 g/dl (IQR: 11.8–8.3), 328.5 U/L (IQR: 237.2–432.7), 111.0 U/ml (IQR: 64.0–186.5) and 35.3 ng/ml (IQR 10.6–198.5), respectively.

A PSA response ≥50% was observed in 46.8% of patients, with a median time to response of 8.3 months (IQR: 2.1−8.3). Baseline characteristics of groups with and without PSA response were well balanced, with the exception of ECOG performance score, hemoglobin and PSA levels, as significantly more patients in the no PSA response group had hemoglobin levels <12 g/dl, higher PSA values and worse ECOG performance scores (p = 0.022, 0.019 and 0.010, respectively). Characteristics of both the groups are detailed in Table 1.

**Table 1.** Patients’ baseline characteristics in the global population and stratified by prostate-specific antigen response.

Characteristic	Global	PSA response	No PSA response	p-value
Patients	124	58	66	−
Age (years):				
− Median	73.4	75.1	74	0.589
− IQR	67.1–80.0	67.84–81.37	64.85–79.15	−
Gleason score:				
− ≥8	52 (41.9)	24 (41.4)	28 (42.4)	0.692
− <8	54 (43.5)	27 (46.5)	27 (40.1)	
Metastasis site:				
− Bone	116 (93.5)	54 (93.1)	62 (93.9)	0.850
− Lymph node	59 (47.6)	29 (50)	30 (45.4)	0.613
− All visceral	22 (17.7)	10 (17.2)	12 (18.2)	0.891
− Lung	25 (20.2)	11 (19.0)	14 (21.2)	0.829
− Hepatic	9 (7.3)	6 (10.3)	3 (4.5)	0.315
Performance status – ECOG:				
− 0–1	113 (91.1)	57 (98.3)	56 (84.8)	0.010
− ≥2	11 (8.9)	1 (1.7)	10 (15.2)	
Previous docetaxel:				
− Yes	47 (37.9)	17 (29.3)	30 (45.5)	0.064
− No	77 (62.1)	41 (70.7)	36 (54.5)	
Primary treated:				
− Yes	58 (46.8)	38 (65.5)	40 (60.6)	0.572
− No	60 (48.4)	20 (34.5)	26 (39.4)	
Time with ADT to mCRPC (months):				
− Median	52.0	48.0	43.0	0.854
− IQR	47.1–66.2	37.5–61.1	42.7–61.7	
Time to mCRPC (months):				
− Median	52	60	45	0.609
− IQR	17–78	24–82	12.25–72	−
Hemoglobin				
− Median, g/dl	12.6	13	12.3	0.022
− IQR, g/dl	11.8–8.27	11.9–13.8	11.5–13.3	−
− ≥12 g/dl	79 (63.7)	40 (69)	39 (59)	0.268
− <12 g/dl	45 (36.3)	18 (31)	27 (41)	
LDH:				
− Median (U/l)	328.5	321	346.5	0.208
− IQR (U/l)	237.2–432.7	222.2–372.5	237.7–495.5	−
− ≥Median	62 (50)	28 (48.3)	33 (50)	0.980
− <Median	62 (50)	30 (51.7)	33 (50)	
ALP:				
− Median (U/ml)	111	99	125	0.275
− IQR (U/l)	64–186.5	58–145.5	70.7–230	−
− ≥Median	33 (26.6)	29 (50)	34 (51.5)	0.128
− <Median	91 (73.4)	29 (50)	32 (48.5)	
Total PSA:				
− Median (ng/ml)	35.3	19.9	68.6	0.019
− IQR (ng/ml)	10.65–198.5	9.9–100.7	18.7–297.7	−
− ≥Median	31 (25)	30 (51.7)	33 (50)	0.120
− <Median	93 (75)	28 (48.3)	33 (50)	
Follow-up, median (months)	11.5	−	−	−

Data represented as n (%) unless otherwise stated.

ADT: Androgen-deprivation therapy; ECOG: Eastern Cooperative Oncology Group; IQR: Interquartile range; mCRPC: Metastatic castration-resistant prostate cancer; PSA: Prostate-specific antigen.

The overall median follow-up was 11.5 months. A total of 73 patients (58.9%) had died by the last follow-up, 24 of which in the group with PSA response and 49 in the group without PSA response. 27 patients remain on AA treatment, with progression being the primary reason for treatment discontinuation.

### Duration of response in patients treated with AA, stratified by PSA response

**Figure 1.**
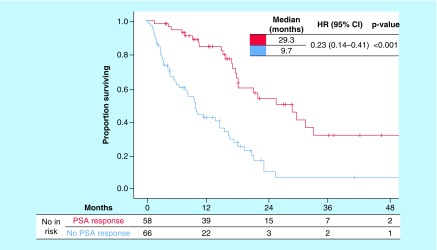
Kaplan–Meier curves of overall survival according to prostate-specific antigen response. HR: Hazard ratio; PSA: Prostate-specific antigen.

Duration of response in terms of PFS is presented in Table 2. Median PFS was significantly longer in the group with PSA response: 17.0 versus 5.3 months in the group without PSA response. This indicates that PSA response resulted in a 77% reduction in the risk of progression compared with having no PSA response (hazard ratio [HR]: 0.23; 95% CI: 0.14−0.39; p < 0.001). PFS Kaplan–Meier curves are shown in Figure 1. In univariate analysis, patients with PSA response, baseline ALP and total PSA below the median, not previously treated with docetaxel and better ECOG performance score were associated with a better PFS (Table 2).

**Table 2.** Prognostic role of baseline characteristics and prostate-specific antigen response: univariate and multivariate analysis for overall survival.

Univariate	Median PFS (months)	HR (95% CI)	p-value
PSA response >50%:			
− Yes	17.0	0.23 (0.14–0.39)	<0.001
− No	5.2	ref.	
Gleason score:			
− <8	12.3	0.71 (0.43–1.20)	0.199
− ≥8	8.2	ref.	
Primary treated:			
− Yes	11.3	0.99 (0.59–1.68)	0.992
− No	9.6	ref.	
Previous docetaxel:			
− No	13.5	0.61 (0.38–0.98)	0.041
− Yes	7.7	ref.	
Visceral metastasis:			
− No	11.3	0.97 (0.49–1.91)	0.934
− Yes	6.2	ref.	
ECOG:			
− 0–1	11.3	0.27 (0.11–0.69)	0.006
− ≥2	3.9	ref.	
ALP:			
− <ULN	12.7	0.48 (0.28–0.81)	0.006
− ≥ULN	6.2	ref.	
Hemoglobin:			
− ≥12 g/dl	12.3	0.89 (0.54–1.45)	0.631
− <12 g/dl	9.2	ref.	
Total PSA:			
− <Median	16.5	0.48 (0.29–0.77)	0.002
− ≥Median	6.7	ref.	
LDH:			
− <Median	9.6	1.03 (0.64–1.65)	0.53
− ≥Median	11.3	ref.	
Multivariate			
PSA response >50%:		0.24 (0.14–0.41)	<0.001
− Yes		ref.	
− No			
Previous docetaxel:			
− Yes		ref.	0.990
− No		0.99 (0.59–1.70)	
ECOG:			
− 0–1		0.35 (0.13–0.95)	0.040
− ≥2		ref.	
ALP:			
− <ULN		0.85 (0.44–1.62)	0.613
− ≥ULN		ref.	
Total PSA:			
− <Median		0.62 (0.35–1.09)	0.099
− ≥Median		ref.	

ECOG: Eastern Cooperative Oncology Group; HR: Hazard ratio; IQR: Interquartile range; OS: Overall survival; PFS: Progression-free survival; PSA: Prostate-specific antigen; ULN: Upper limit of normal.

After adjusting for significant parameters at univariate analysis, a ≥50% reduction in PSA remained predictive of better PFS (HR: 0.24; 95% CI: 0.14–0.41; p < 0.001). A better ECOG performance score (HR: 0.35; 95% CI: 0.13–0.95; p = 0.040) also remained an independent factor for PFS benefit.

### Survival in patients treated with AA, stratified by PSA response

**Figure 2.**
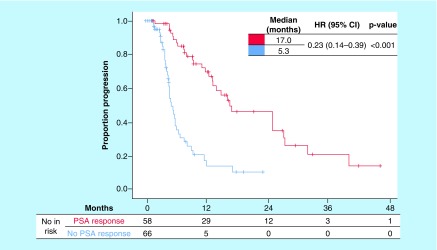
Kaplan–Meier curves of progression-free survival according to prostate-specific antigen response. HR: Hazard ratio; PSA: Prostate-specific antigen.

Median OS by Kaplan–Meier analysis (Figure 2) was significantly longer for patients with PSA response compared with patients without PSA response (29.3 vs 9.7 months). This indicates that having a PSA response resulted in a 71% reduction in the risk of death compared with not having a PSA response (HR: 0.29; 95% CI: 0.18–0.48; p < 0.001). OS analysis is shown in Table 3.

**Table 3.** Prognostic role of baseline characteristics and prostate-specific antigen response: univariate and multivariate analysis for progression-free survival.

Univariate	Median OS (months)	HR (95% CI)	p-value
PSA response >50%			
– Yes	29.3	0.29 (0.18–0.48)	<0.001
– No	9.7	ref.	
Gleason score			
– <8	14.1	0.87 (0.52–1.46)	0.603
– ≥8	11.1	ref.	
Primary treated			
– Yes	19.6	0.54 (0.34–0.87)	0.011
– No	10.4	ref.	
Previous docetaxel			
– No	21.5	0.49 (0.31–0.77)	0.002
– Yes	10.5	ref.	
Visceral metastasis			
– No	18.1	0.55 (0.31–0.95)	0.033
– Yes	8.1	ref.	
ECOG			
– 0–1	18.1	0.21 (0.10–0.43)	<0.001
– ≥2	3	ref.	
ALP			
– <ULN	18.2	0.52 (0.32–0.84)	0.008
– ≥ULN	10.5	ref.	
Hemoglobin			
– ≥12 g/dl	18.4	0.54 (0.34–0.87)	0.01
– <12 g/dl	10.5	ref.	
Total PSA			
– <Median	25.8	0.30 (0.18–0.48)	<0.001
– ≥Median	9.5	ref.	
LDH			
– <Median	18.4	0.68 (0.42–1.08)	0.1
– ≥Median	14.6	ref.	
**Multivariate**			
PSA response >50%			
– Yes		0.19 (0.10–0.38)	<0.001
– No		ref.	
Primary treated			
– Yes		0.47 (0.27–0.80)	0.006
– No		ref.	
Previous docetaxel			
– 0		0.69 (0.41–1.14)	0.148
– 1		ref.	
Visceral metastasis			
– No		0.64 (0.36–1.15)	0.137
– Yes		ref.	
ECOG			
– 0–1		0.50 (0.21–1.18)	0.112
– ≥2		ref.	
ALP			
– <ULN		0.73 (0.40–1.31)	0.291
– ≥ULN		ref.	
Hemoglobin			
– ≥12 g/dl		0.62 (0.34–1.14)	0.121
– <12 g/dl		ref.	
Total PSA			
– <Median		0.35 (0.20–0.59)	<0.001
– ≥Median		ref.	

ECOG: Eastern Cooperative Oncology Group; HR: Hazard ratio; IQR: Interquartile range; OS: Overall survival; PFS: Progression-free survival; PSA: Prostate-specific antigen; ULN: Upper limit of normal.

In univariate analysis, PSA response, treatment for the primary tumor, absence of visceral metastases, absence of previous chemotherapy, better ECOG performance score, baseline hemoglobin ≥12 g/dl and baseline PSA and ALP below the median were associated with better OS (Table 3). After adjusting for significant factors at univariate analysis, multivariate analysis revealed that a ≥50% reduction in PSA remained predictive of better survival prognosis (HR: 0.19; 95% CI: 0.10–0.38; p < 0.001), as well as baseline PSA below the median (HR: 0.35; 95% CI: 0.20–0.59; p < 0.001) and previous treatment for the primary tumor (HR: 0.47; 95% CI: 0.27–0.80; p = 0.006; Table 3).

## Discussion

With the growing number of therapeutic options capable of extending survival in mCRPC patients, there is a need for biomarkers that can simultaneously guide treatment decisions and predict which patients will benefit the most from new treatments as AA. Although this is true for all tumors, it is particularly relevant for prostate cancer, due to factors related with disease heterogeneity and difficulty in assessing disease in bone and interpreting the clinical significance of post-therapy PSA changes in different settings.

In this cohort of mCRPC patients treated with AA, PSA response was associated with both OS and PFS benefit. After adjusting for other clinically relevant and statistically significant baseline characteristics, this factor remained prognostic for increased survival and predictive of treatment response. The development of new surrogate markers for clinical outcomes is becoming increasingly important with the emergence of multiple lines of treatment with survival benefit for mCRPC patients. With OS being the gold-standard end point in Phase III clinical trials, the multiple sequencing treatment options that are becoming available will predictably disturb the analysis of these end points. Therefore, surrogates for OS can provide clinicians with data that better inform them throughout patient treatment, allowing for more robust go/no-go decisions. Defining a new surrogate end point requires meeting several criteria defined by Prentice criteria or by the proportion of treatment effect, as previously explained [11].

PSA remains a questionable surrogate for survival in late-stage prostate cancer. PCWG2 states that the clinical significance of a post-therapy PSA decline remains controversial and advises against its early use. In fact, an increase in serum PSA, or ‘flare’, may occur in some patients before they experience a subsequent and sometimes significant PSA decline. This is why PCWG2 recommends securing a sufficiently large drug exposure window and avoid relying on serum PSA decline as a surrogate for clinical benefit. Perhaps even more importantly, the group advises not to interpret a rise in serum PSA as progression and early withdrawing a therapy from which the patient may benefit [12,13]. Drugs targeting androgen receptor (AR) signaling, such as AA, may have a different association to an early PSA decrease, since PSA is a pharmacodynamic biomarker of androgen receptor signaling in absence of aberrations on the PSA promoter or key regulators of PSA production and secretion [15].

Some studies reported that patients with mCRPC who experienced ≥50% PSA decline from baseline had improved survival compared with those who did not. Additional studies, like the retrospective analysis of PSA decline in the SWOG 99-16 trial, which compared docetaxel plus estramustine to mitoxantrone plus prednisone, indicated that a PSA reduction of at least 20−40%, a 2-month PSA decline of 30% and PSA velocity of decline at 2 and 3 months after therapy satisfy the stringent requirements of statistical surrogacy for prediction of OS. The TAX327 trial is another such example, showing that a ≥30% PSA decline after docetaxel treatment fulfilled the Prentice criteria for surrogacy. It is worth mentioning that most of these results were observed in patients treated with first-line chemotherapy, and the evidence supporting PSA decline as a surrogate for OS across multiple drug classes is scarce and conflicting [11,12,15].

Although this study showed a potential role for PSA response as a predictive and prognostic factor for response in mCRPC, it has some limitations, the primary being its small sample size and retrospective nature. Further validation of its results is, therefore, required to assist in implementation of the best clinical practice and allow patients unlikely to benefit from AA to switch to another treatment option. Additionally, validation of PSA as a biomarker in mCRPC has the potential to help identify patient subgroups that may benefit the most from AA, maximizing cost–effectiveness of this treatment [16]. Finally, it should be noted that new molecules, such as Radium-223, with proven therapeutic effect in mCRPC patients, do not usually induce a PSA response, therefore other biomarkers should be additionally investigated.

## Conclusion

In the present study, PSA response was an independent predictive and prognostic factor for survival outcomes, with PSA response being associated with response duration and survival benefit in patients treated with AA. Biochemical markers are becoming increasingly important as response and prognostic surrogates, especially for diseases difficult to evaluate radiographically (>90% bone metastases). Further prospective studies in larger patient populations are required to confirm these findings.

## Future perspective

Further advances are expected to occur in this area in upcoming years, with new and more effective treatment options. Associated with that, more than prognostic also predictive biomarkers will be important to investigate, as they can potentially predict response before treatment's start, avoiding toxicities and maximizing treatment cost–effectiveness.

**Summary points**

***Background:***

- Biomarkers with predictive and prognostic value to assist in patient selection and treatment guidance in metastatic castration-resistant prostate cancer (mCRPC) are lacking;
- Studies have shown that patients achieving a 50% prostate-specific antigen (PSA) reduction have better survival outcomes compared with those who do not. Yet, in the era of new hormonal therapies, as abiraterone acetate (AA), the role of PSA as a surrogate endpoint for overall survival (OS) remains unclear;
- The aim of this study was to evaluate prostate-specific antigen response (PSAr) as a prognostic factor in patients treated with AA.

***Methods:***

- Clinical data of patients with mCRPC treated with AA were retrospectively evaluated;
- The primary objective was to evaluate association of PSAr – defined as a 50% or higher reduction in the initial PSA value – with OS and PFS;
- Median OS and PFS correlations were obtained using Kaplan-Meier method and controlling with a multivariate Cox regression model.

***Results:***

- A total of 124 patients were treated with AA between January 2013 and December 2017;
- 58 patients (48.6% achieved PSAr, with a median time until PSAr of 8.3 months (IQR: 5.25–19.19);
- Median OS was 29.3 months for patients achieving PSAr vs 9.7 months for patients not achieving PSAr (HR: 0.29; 95% CI: 0.18–0.48; p < 0.001);
- PFS was 17 months for patients with PSAr and 5.2 months for patients without PSAr (HR: 0.23; 95% CI: 0.14–0.39; p < 0.001);
- In multivariate analysis, PSAr was confirmed as an independent prognostic factor for OS (HR: 0.19; 95% CI: 0.10–0.38; p < 0.001) and PFS (HR: 0.24; 95% CI: 0.14–0.41; p < 0.001).

***Conclusions and future perspectives:***

- In this retrospective analysis, PSAr correlated with significantly better PFS and OS;
- Biochemical markers are becoming increasingly relevant as surrogates for response and prognosis, especially in diseases difficult to evauate radiographically;
- These findings require fguther validation in larger prospective studies.
